# Patterns and Prognostic Value of Lymph Node Metastasis on Distant Metastasis and Survival in Nasopharyngeal Carcinoma: A Surveillance, Epidemiology, and End Results Study, 2006–2015

**DOI:** 10.1155/2019/4094395

**Published:** 2019-11-29

**Authors:** Yali Xu, Taoyuan Huang, Liqin Fan, Wei Jin, Xiaoming Chen, Jinhai Chen

**Affiliations:** ^1^Department of Otolaryngology, The Second Affiliated Hospital of Guangzhou Medical University, Guangzhou 510260, China; ^2^Department of Dermatology, Dermatology Hospital of Southern Medical University, Guangzhou 510091, China

## Abstract

This study was conducted to identify factors associated with lymph node (LN) metastasis in nasopharyngeal carcinoma (NPC) patients, analyze node distribution patterns, and explore the prognostic value of the LN metastasis level for survival. We included 2994 patients with primary NPC diagnosed between 2006 and 2015 with information in the Surveillance, Epidemiology, and End Results (SEER) database. Patients' demographic and clinicopathologic features were compared according to LN status using chi-squared tests. The 5-year overall survival (OS) and cancer-specific survival (CSS) rates were calculated by the Kaplan–Meier method, and the differences were estimated by log-rank tests. Multivariate Cox proportional hazard models were used to evaluate independent risk factors for OS and CSS. Logistic regression was used to evaluate the risk of each LN metastasis category for distant metastasis. There were 695 patients in the N0 stage and 2299 with LN metastasis (classified as stage N1, N2, or N3). The overall incidence of LN metastasis was 76.8%. Sex and T stage were not associated with LN metastasis. Older patients had a significantly worse 5-year OS and CSS than younger patients. In terms of histologic type, keratinizing squamous cell carcinoma had the lowest 5-year OS and CSS at 48.2% and 53.8%, respectively. The most common nodal involvement level was II (65.9%), followed by III (29.1%), V (25.6%), I (17.6%), IV (15.7%), and retropharynx (13.5%). The skip metastasis rate was 5.7% (130/2299). Patients with only level II metastasis (classified as level 2) was the most common category, accounting for 30%. Compared to level 2, patients with only level I (classified as level 1) had an OR of 2.101 (95% CI: 1.090–4.047, *P*=0.027) for distant metastasis, patients with simultaneous levels II, III, IV, and V (classified as levels 2345) had the highest OR of 4.064 (95% CI: 2.155–7.666, *P* < 0.001) for distant metastasis, and level 24 had an OR of 3.003 (95% CI: 1.074–8.395, *P*=0.036) for distant metastasis. In survival analysis, levels 235 had a significant HR of 1.708 (95% CI: 1.089–2.678, *P*=0.020) for CSS compared to level 2 after adjustment for age, sex, race, histology, TNM (tumor, node, and metastasis) stage, and treatment.

## 1. Introduction

Nasopharyngeal carcinoma (NPC) is a rare cancer arising from the nasopharynx epithelium. Globally, the incidence rate of NPC is less than 1 case per 100,000 person-years [1]; however, it is as high as 20 to 40 per 100,000 persons in endemic regions such as Southeast Asia and North Africa [2, 3].

Among head and neck cancers, NPC has the highest preponderance for regional lymph node (LN) metastasis [4]. Due to the region's vast submucosal avalvular lymph capillary network, the incidence of LN metastasis is as high as 70%. With the development of advanced imaging methods such as magnetic resonance imaging (MRI) and positron emission tomography/computed tomography (PET/CT), LN metastasis is detected more easily and accurately. LN metastasis patterns are significant predictors of distant metastasis and have prognostic effects on overall and distant metastasis-free survival [5, 6]. To our knowledge, most studies have focused on the distribution probabilities in each level [7–9]; however, there is limited information about prognostic value of LN metastasis category in distant metastasis and survival.

Radiotherapy is the primary and only curative treatment for NPC. Intensity-modulated radiotherapy (IMRT) is the preferred method as it provides the therapeutic benefit of dose escalation to tumor tissue with reduced toxicity to normal tissues [10]. Compared to other treatments, IMRT has reduced radiation-related toxicity and can improve NPC patient-reported quality of life [11]. Morbidities of IMRT including dermatitis, odynophagia, neck fibrosis, and brachial plexopathy are associated with the larger field of the cervical LN target volumes [12]. The definition of the appropriate clinical target volume for cervical LN in NPC is still a matter of debate [13].

The Surveillance, Epidemiology, and End Results (SEER) program is a well-established and extensive collection of data from cancer registries throughout the United States [14]. It includes almost all cancer incidence, prevalence, demographic, and clinicopathologic datasets. In this study, we sought to investigate factors associated with the incidence of LN metastasis and calculate hazard ratios (HRs), overall survival (OS), and cancer-specific survival (CSS) in 2994 NPC patients. We also analyzed LN distribution patterns and evaluated the LN metastasis categories, which may predict the prognosis of 1884 NPC patients with complete information of LN involvement level.

## 2. Materials and Methods

### 2.1. Study Population

The SEER program collects data from 18 original registries that cover approximately 27.8% of the U.S. population. We used SEER*∗*Stat version 8.3.5 software to extract the data from the SEER database. We included patients who were diagnosed with only primary NPC from 2005 to 2016 using the International Classification of Diseases O-3 site recodes C11.0–C11.9 and histologic types 8020, 8021, 8070–8073, 8082, and 8083. Patients were excluded if the type of reporting source was from autopsy or death certificate. We also excluded those without complete stage information. Ultimately, 2994 patients were included in this study.

Incidence rates were age-adjusted to the standard U.S. 2000 population. The annual percent change (APC) in incidence was also calculated with a 1-year end-point, and significance testing was performed with weighted least squares.

Age was grouped into five categories: <40, 40–49, 50–59, 60–69, and >70 years. Racial classifications were Asian/Pacific Islander, White, Black, American Indian/Alaska Native, and unknown. Based on the World Health Organization (WHO) classification scheme, histology was divided into keratinizing squamous cell carcinoma (KSCC, SEER codes 8070, 8071; squamous cell carcinoma), nonkeratinizing carcinoma (NKSCC, SEER codes 8020, 8021, 8072, 8073, and 8082; undifferentiated, anaplastic, large- and small-cell nonkeratinizing carcinomas, and lymphoepithelial carcinoma), and basaloid squamous cell carcinoma (BSCC, SEER code 8083; basaloid squamous cell carcinoma). The AJCC 6th (diagnosis years from 2006 to 2009) and 7th (diagnosis years from 2010 to 2016) editions were used for staging. Based on a study that demonstrated superiority of the 7th edition [15], we reclassified the T2a category in the 6th edition to T1, and T2b to T2. LN metastasis levels were coded based on the Collaborative Stage Site-Specific Factors (CS Site-Specific Factors 3 and 4) including levels I, II, III, IV, V, and retropharyngeal (rp) LNs. A patient with only level I metastasis was classified as level 1, the patient with level I and II was classified as levels 12, etc. The patient that skipped a level would be classified accordingly (e.g., levels 124).

### 2.2. Statistical Analysis

Comparisons of categorical variables between patients with and without LN metastasis were performed using chi-squared tests. The 5-year OS rates and CSS rates were calculated by Kaplan–Meier analysis, and survival differences were estimated with log-rank tests. Logistic regression was used to evaluate the risk LN metastasis category for distant metastasis. We calculated HRs and their 95% confidence intervals (CIs) using multivariate Cox proportional hazards regression models to evaluate the effects of LN metastasis compound category on CSS. All analyses were conducted using SPSS version 25 statistical software (IBM Corporation). *P* < 0.05 was considered statistically significant.

## 3. Results

### 3.1. Incidence Analysis

**Figure 1:**
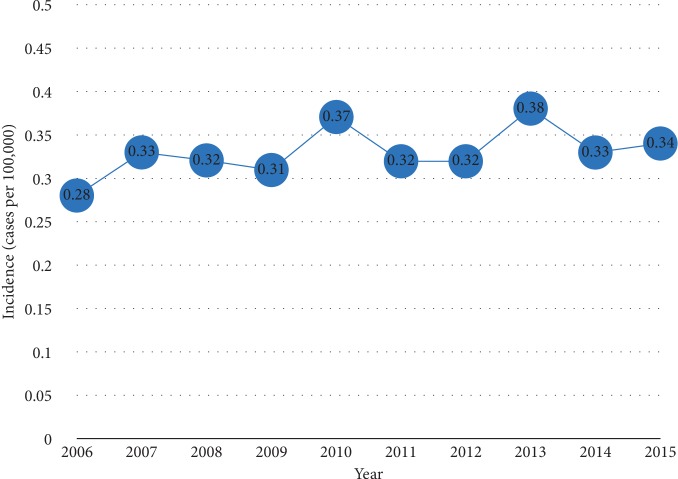
Incidence trends for NPC, 2006–2015.

We collected age-adjusted incidence data from the original 18 registry sites in the SEER database. The overall incidence of NPC during 2006–2015 was 0.33 patient per 100,000 population (0.49/100,000 for males and 0.19/100,000 for females). The annual overall incidence rates for the study period are shown in Figure 1. They remained stable (APC 1.45, 95% CI: −0.59–3.53, *P*=0.14) from 2005 to 2016.

### 3.2. Patient Baseline Characteristics

A total of 2994 NPC patients diagnosed between 2006 and 2015 were enrolled, including 2139 (71.4%) males and 855 (28.6%) females. The male-to-female ratio was 2.5 : 1. The median age at diagnosis was 54 (range, 8–99) years. Most patients were White (47.4%), followed by Asian/Pacific Islander (37.7%), Black (12.4%), American Indian/Alaska Native (1.7%), and unknown (0.8%). Among NPC subtypes, NKSCC had the highest incidence at 55.6%, compared with KSCC (43.0%) and BSCC (1.4%). As for the TNM stage, 1610 (53.8%) patients were T1 + T2, 634 (21.2%) were T3, and 750 (25.1%) were T4; 1684 (56.2%) were N0 + N1, 906 (30.3%) were N2, and 404 (13.5%) were N3; 2695 (90.0%) were M0, and 299 (10.0%) were M1.

### 3.3. Factors Associated with LN Metastasis

Correlations between NPC patient characteristics and LN metastasis status are shown in Table 1. Younger patients (age at diagnosis <40 years) had a significantly higher percentage of LN metastasis than older patients (85.6% vs. 82.4%, 76.9%, 71.3% for 40–49, 50–59, 60–69, and >70 years, respectively, *P* < 0.001). With regard to race, the American Indian/Alaska Native group had the highest rate of LN metastasis (82.7% vs. 80.2%, 74.0%, and 77.6% in Asian/Pacific Islander, White, Black, and American Indian/Alaska Native, respectively, *P*=0.002). LN metastasis was less common in patients with BSCC compared to other subtypes (60.5% vs. 75.1% and 78.5% for the KSCC and NKSCC subtypes, respectively, *P*=0.004). Surprisingly, no significant correlation was found for the incidence of LN metastasis regarding sex or T stage (both *P* > 0.05).

### 3.4. Influence of N Stage on 5-Year OS and CSS Rates and Multivariate Analysis Findings

The 5-year OS and CSS rates for the entire population were 59.4% and 63.7%, respectively. As shown in Table 2, the 5-year OS and CSS were significantly longer in the N0 + N1 group than in other groups (OS: 62.8% vs. 57.5% and 47.4% for N2 and N3 stages, respectively, *P* < 0.001; CSS: 67.7% vs. 60.9% and 51.1% for N2 and N3 stages, respectively, *P* < 0.001). Younger patients (<40 years) had significantly longer OS and CSS than older patients (OS: 76.6% vs. 67.5%, 59.9%, 49.8%, and 36.1% for 40–49, 50–59, 60–69, and >70 years, respectively, *P* < 0.001; CSS: 78.5% vs. 68.8%, 63.8%, 54.4%, 47.4% for 40–49, 50–59, 60–69, and >70 years, respectively, *P* < 0.001). Female patients had significantly longer OS and CSS than male patients (OS: 64.1% vs. 57.6%, *P*=0.002; CSS: 68.9% vs. 61.7%). In terms of race, after excluding patients categorized as unknown (*n* = 24), Asian/Pacific Islanders had the highest OS and CSS rates at 67.3% and 70.2%, respectively, while American Indian/Alaska Native had the lowest OS and CSS at 43.8% and 45.3%, respectively. Among histologic subtypes, KSCC had the lowest OS and CSS (OS: 48.2% vs. 67.9% and 61.8% in NKSCC and BSCC, respectively, *P* < 0.001; CSS: 53.8% vs. 71.1% and 66.8% in NKSCC and BSCC, respectively, *P* < 0.001). Patients recommended to undergo surgery had better 5-year OS and CSS rates than those not/unknown recommended (OS: 65.9% vs. 58.2%, *P*=0.006; CSS: 71.5% vs. 62.3%, *P*=0.004). A total of 846 patients received radiotherapy, and their 5-year OS and CSS were better than that in patients without radiotherapy (OS: 66.9% vs. 56.3%, *P* < 0.001; CSS: 71.8% vs. 60.4%, *P* < 0.001). Finally, patients undergoing chemotherapy had higher 5-year OS and CSS rates than those without chemotherapy (OS: 62.1% vs. 45.6%, *P* < 0.001; CSS: 65.9% vs. 52.4%, *P* < 0.001). We performed multivariate analysis using Cox proportional hazards regression modeling and found that age, sex, race, histology type, TNM stage, surgery, radiotherapy, and chemotherapy were significant independent prognostic factors for OS and CSS. Compared with the N0 + N1 group, N2 patients had a HR of 1.311 (95% CI: 1.135–1.514, *P* < 0.001) for OS and the N3 group had a HR of 1.625 (95% CI:1.357–1.945, *P* < 0.01) for OS. In addition, N2 patients had a HR of 1.351 for CSS (95% CI: 1.156–1.580, *P* < 0.001) and the N3 group had a HR of 1.630 for CSS (95% CI:1.342–1.979, *P* < 0.01).

### 3.5. Incidence and Distribution of LN Metastasis

A total of 2299 (76.8%) patients with lymphadenopathy (classified as N1, N2, or N3) were enrolled. The distributions were as follows: 404 (17.6%) cases in level I, 1515 (65.9%) in level II, 670 (29.1%) in level III, 360 (15.7%) in level IV, 588 (25.6%) in level V, and 311 (13.5%) in the rp level. Eighty-one cases presented with level III LN involvement without level II LN involvement. We also identified 49 cases with level IV LN involvement without level II and III involvement. The skip metastasis rate was 5.7% (130/2299).

### 3.6. The Risk LN Metastasis Categories for Distant Metastasis

After excluding 415 patients with incomplete LN metastasis level information, 1884 patients were ultimately unrolled. Level 2 is the most commonly-seen LN metastasis category in NPC patients with LN involvement (30.0% of 1884 eligible patients). This was followed by levels 23 (8.5%), level 1 (5.5%), level 5 (5.3%), levels 25 (5.3%), levels 12 (4.0%), levels 2345 (3.9%), levels 235 (3.8%), levels 2rp (3.2%), levels 234 (3.1%), level rp (2.4%), level 3 (2.2%), levels 23rp (2.1%), levels 24 (1.5%), and others (19.4%).  Table 3 shows that, taken level 2 as reference, patients with level 1 had an OR of 2.101 (95% CI: 1.090–4.047, *P*=0.027) for distant metastasis, levels 2345 had the highest OR of 4.064 (95% CI: 2.155–7.666, *P* < 0.001) for distant metastasis, and levels 24 had an OR of 3.003 (95% CI: 1.074–8.395, *P*=0.036) for distant metastasis after adjustment for age, sex and race, which were also statistically significant in univariate analysis.

### 3.7. Prognostic Values of LN Metastasis Categories for CSS in Patients with NPC

As shown in Table 4, levels 235 had a statistically significant HR of 1.708 (95% CI: 1.089–2.678, *P*=0.020) for CCS compared to level 2 after adjustment for age, sex, race, histology, TNM stage, and treatment strategies, while it is not statistically significant before (*P*=0.056). Level 3 had a significant HR of 1.709 (95% CI: 1.017–2.873, *P*=0.043) in univariate analysis; however, it reduced to 1.602 (95% CI: 0.938–2.737) with no statistical significance (*P*=0.084).

## 4. Discussion

NPC is a malignant tumor of the epithelial tissue, and its incidence and mortality vary by race, sex, age, and country [16]. In our study, the overall U.S. incidence of NPC from 2006–2015 was 0.33 per 100,000 population. Males are two to three times more likely to develop the disease than women, and the peak age of disease occurrence is between 50 and 60 years [1], which was similar to our results (male-to-female ratio of 2.6 : 1). Although substantial declines in NPC incidence rates have been reported in nearly all Asian studies [16], we found that the U.S. incidence remained stable in our study.

In a retrospective cohort study by Challapalli and colleagues, Asian/Pacific patients had the highest NPC incidence rate, but they also had the highest 5-year CSS rate, while American Indian/Alaska Native had the lowest survival [17]. Lee and Ko analyzed epidemiologic patterns and survival rates for NPC patients using the SEER statistical program and found that poor survival was associated with older age, male sex, and KSCC histology [18]. Radiotherapy is the primary and only curative treatment for NPC [19]. A meta-analysis examining the effects of concomitant chemotherapy with and without adjuvant chemotherapy in NPC demonstrated improvement in OS [20]. Sun et al. performed a retrospective population-based analysis and provided evidence of a positive impact of surgery on survival in NPC [21]. Recent treatment advances have improved clinical outcomes, resulting in longer survival for NPC patients. Our results also show that the 5-year OS was longer for patients that were recommended surgery or received radiotherapy and chemotherapy compared to those that did not receive treatment.

NPC usually presents with LN metastasis, 36%–45% of NPC patients present with the initial symptoms of neck lymphadenectasis, and 60%–90% have cervical LN metastasis at NPC diagnosis [22]. A meta-analysis by Ho et al. revealed that 85% of 2920 Chinese NPC patients staged using MRI had LN involvement at diagnosis [23], which is higher than our result (76.8%). We found that sex and T stage were not associated with LN metastasis, which is consistent with the report by Tomita and colleagues [24]. They found that LN metastasis was not correlated with sex differences including lifestyle behaviors and biologic traits (e.g., sex hormones). Their study divided patients into two groups (≤60 vs. >60 years) and found that age was not significantly correlated with the incidence of LN metastasis. However, we stratified age into five groups and found that younger patients (<40 years) had a significantly higher rate of LN metastasis than older patients. The effect of age on the LN metastasis remains disputed. In terms of T stage, Lv et al. examined 1067 patients with LN involvement in T1-T2 and T3-T4 groups and found no significant correlation between T stage and nodal involvement [25]. This suggests that T and N stages are dependent on tumor biological behaviors. Therefore, it is appropriate to make clinical treatment decisions based on the T and N stages, rather than the total stage. The WHO pathological type of NPC has different clinical characteristics and prognostic information; it is significantly associated with the incidence of cervical LN metastasis. One retrospective study found that patients with NKSCC showed a higher incidence of LN metastasis than patients with KSCC [24], which was in accordance with our results. Another retrospective population-based analysis reported an LN metastasis rate for BSCC to be 53.7% [26], which was lower than our rate of 60.5%.

The actual distributions of nodal metastasis as described in terms of LN level differs between studies. Most reported that level II and retropharyngeal LNs were the first affected by metastasis, with overall probabilities of 60%–80% [23, 25]. Chua et al. found a 29.1% rate of retropharyngeal LN involvement [27]. Our results showed that the probabilities of level II and retropharyngeal LN involvement were 65.9% and 13.5%, respectively. These discrepancies may be attributed to variable detection methods and diagnostic standards. Retropharyngeal LN metastases are isodense and contiguous with the primary tumor and thus may not be identified as a separate mass on CT [7]. The superior soft-tissue contrast of MRI appears to be a better option for LN identification. These factors may have influenced the low incidence of retropharyngeal LN involvement compared to other reports. Several recent studies have reported that LN metastasis in NPC occurs in an orderly fashion from the higher to lower level LNs, and skip metastasis is rare, ranging from 0.5% to 7.9% [28, 29]. The overall frequency of skip metastasis in our series was 5.7%.

Metastatic nodes from NPC have been associated with poor outcome, especially for cases with bulky nodes (>6 cm), necrosis, or nodes in the lower neck [30, 31]. Compared with the N0 + N1 group, the N2 group had a HR of 1.311 (95% CI: 1.135–1.514, *P* < 0.001) for OS and 1.351 for CSS (95% CI: 1.156–1.580, *P* < 0.001), while the N3 group had a HR of 1.625 (95% CI: 1.357–1.945, *P* < 0.01) for OS and 1.630 for CSS (95% CI: 1.342–1.979, *P* < 0.01). Although nodal metastases are associated with poorer prognoses, the recommendations for treatment, the correlation between LN metastases and distant metastases, and how tumor cells enter the bloodstream via LNs still remained debate. Two recent studies in mouse models revealed that distant metastasis is driven by direct invasion of tumor cells into LN blood vessels [32] rather than through the thoracic duct [33]. To examine the influence of LN metastasis on distant metastasis, we classified eligible patients into LN metastasis categories (e.g., level 2, levels 23, level 1, level 5, and levels 25).To our knowledge, this is the first population-based analysis to use LN metastasis compound category to provide evidence of the impacts of LN metastasis on distant metastasis and survival in NPC. Taking level 2 as reference, level 1 had an OR of 2.101 (95% CI: 1.090–4.047, *P*=0.027) and levels 24 had an OR of 3.003 (95% CI: 1.074–8.395, *P*=0.036) for distant metastasis after adjustment for age, sex, and race. Notably, levels 2345 category had the highest OR of 4.064 (95% CI: 2.155–7.666, *P* < 0.001) for distant metastasis. In survival analysis, levels 235 had a statistically significant HR of 1.708 (95% CI: 1.089–2.678, *P*=0.020) for CSS compared to level 2 after adjustment for age, sex, race, histology, TNM stage, and treatment strategies, while it was not statistically significant before (HR = 1.518, 95% CI: 0.989–2.329, *P*=0.056).

Our results should be interpreted with caution due to limitations associated with use of the SEER database. First, the database does not specify the detection methods or diagnostic criteria of LN involvement. Second, we did not include well-known prognostic factors such as nodal necrosis, nodal extracapsular spread, or laterality. It remains unknown if incorporating these factors would alter the study conclusions. Nevertheless, our findings provide evidence of the impact of LN metastasis category on distant metastasis and survival in NPC.

## 5. Conclusions

The incidence of LN involvement in NPC patients differed by age, race, histologic type, and M stage but not sex or T stage. Taking level 2 as reference, level 1, levels 2345, and levels 24 categories had significantly higher risks for distant metastasis after adjustment for age, sex, and race. Levels 235 had a statistically significant HR of 1.708 for CSS compared to level 2 after adjustment for age, sex, race, histology, TNM stage, and treatment.

## Figures and Tables

**Table 1:** Comparison of clinicopathologic demographics between 2994 NPC patients according to LN involvement.

Characteristics	LN involvement^a^			*P*
	N0 (*n*, %)	N1–3 (*n*, %)	Total number	
Age group (years)				
<40	66 (14.4)	393 (85.6)	459	<0.001
40–49	113 (17.6)	528 (82.4)	641	
50–59	204 (23.1)	681 (76.9)	885	
60–69	173 (28.7)	429 (71.3)	602	
>70	139 (34.2)	268 (65.8)	407	

Sex				
Male	491 (23.0)	1648 (77.0)	2139	0.596
Female	204 (23.9)	651 (76.1)	855	

Race^b^				
Asian/Pacific Islander	223 (19.8)	905 (80.2)	1128	0.002
White	369 (26.0)	1051 (74.0)	1420	
Black	83 (22.4)	287 (77.6)	370	
American Indian/Alaska Native	9 (17.3)	43 (82.7)	52	

Histology				
KSCC	320 (24.9)	966 (75.1)	1286	0.004
NKSCC	358 (21.5)	1307 (78.5)	1665	
BSCC	17 (39.5)	26 (60.5)	43	

T stage^c^				
T1 + T2	363 (22.5)	1247 (77.5)	1610	0.479
T3	146 (23.0)	488 (77.0)	634	
T4	186 (24.8)	564 (75.2)	750	

M stage				
M0	655 (24.3)	2040 (75.7)	2695	<0.001
M1	40 (13.4)	259 (86.6)	299	

BSCC, basaloid squamous cell carcinoma; LN, lymph node; KSCC, keratinizing squamous cell carcinoma; M, distant metastasis; N, node; NKSCC, nonkeratinizing carcinoma; T, tumor. ^a^N0, none; N1, unilateral cervical and/or unilateral or bilateral retropharyngeal node(s), ≤6 cm in greatest dimension, above supraclavicular fossa; N2, bilateral cervical node(s), ≤6 cm in greatest dimension, above supraclavicular fossa; N3, N3a: >6 cm in greatest dimension, above supraclavicular fossa; N3b: in supraclavicular fossa. ^b^24 cases were missing. ^c^T1, nasopharynx, oropharynx, or nasal cavity; T2, parapharyngeal extension; T3, bony structures and/or paranasal sinuses; T4, intracranial extension and/or cranial nerves, hypopharynx, orbit, or infratemporal fossa/masticatory space.

**Table 2:** Kaplan–Meier analyses for 5-year OS and CSS rates and multivariate analyses using Cox proportional hazards regression model in all 2994 NPC patients.

Characteristics	5-year OS		Cox proportional HR		5-year CSS		Cox proportional HR	
	Rate (%)	*P*	HR (95% CI)	*P*	Rate (%)	*P*	HR (95% CI)	*P*
Age group (years)								
<40	76.6	<0.001	1		78.5	<0.001	1	
40–49	67.5		1.394 (1.072, 1.812)	0.013	68.8		1.392 (1.057, 1.834)	0.019
50–59	59.9		1.858 (1.455, 2.373)	<0.001	63.8		1.768 (1.366, 2.290)	<0.001
60–69	49.8		2.422 (1.880, 3.120)	<0.001	54.4		2.189 (1.672, 2.866)	<0.001
>70	36.1		3.812 (2.939, 4.945)	<0.001	47.4		3.036 (2.289, 4.028)	<0.001

Sex								
Male	57.6	0.002	1		61.7	0.002	1	
Female	64.1		0.851 (0.738, 0.980)	0.026	68.9		0.843 (0.721, 0.986)	0.032

Race^a^								
Asian/Pacific Islander	67.3	<0.001	1		70.2	<0.001	1	
White	55.0		1.476 (1.272, 1.712)	<0.001	60.2		1.410 (1.201, 1.657)	<0.001
Black	51.8		1.610 (1.315, 1.971)	<0.001	57.7		1.505 (1.209, 1.874)	<0.001
American Indian/Alaska Native	43.8		1.717 (1.117, 2.638)	0.014	45.3		1.719 (1.095, 2.698)	0.018

Histology								
KSCC	48.2	<0.001	1		53.8	<0.001	1	
NKSCC	67.9		0.596 (0.523, 0.679)	<0.001	71.1		0.582 (0.505, 0.670)	<0.001
BSCC	61.8		0.494 (0.285, 0.858)	0.012	66.8		0.491 (0.270, 0.895)	0.020

T stage^b^								
T1 + T2	67.5	<0.001	1		72.0	<0.001	1	
T3	53.4		1.785 (1.525, 2.089)	<0.001	57.4		1.877 (1.581, 2.227)	<0.001
T4	46.5		2.178 (1.878, 2.527)	<0.001	50.4		2.262 (1.924, 2.659)	<0.001

N stage^c^								
N0 + N1	62.8	<0.001	1		67.7	<0.001	1	
N2	57.5		1.311 (1.135, 1.514)	<0.001	60.9		1.351 (1.156, 1.580)	<0.001
N3	47.4		1.625 (1.357, 1.945)	<0.001	51.1		1.630 (1.342, 1.979)	<0.001

M stage								
M0	63.5	<0.001	1		68.1	<0.001	1	
M1	23.5		2.888 (2.463, 3.386)	<0.001	25.5		3.204 (2.711, 3.786)	<0.001

Surgery								
Recommended	65.9	0.006	1		71.5	0.004	1	
Not recommended/unknown	58.2		1.274 (1.035, 1.568)	0.022	62.3		1.261 (1.001, 1.588)	0.049

Radiotherapy								
Yes	66.9	<0.001	1		71.8	<0.001	1	
No	56.3		1.230 (1.044, 1.449)	0.013	60.4		1.297 (1.082, 1.554)	0.005

Chemotherapy								
Yes	62.1	<0.001	1		65.9	<0.001	1	
No/unknown	45.6		2.437 (2.083, 2.850)	<0.001	52.4		2.461 (2.071, 2.925)	<0.001

BSCC, basaloid squamous cell carcinoma; CI, confidence interval; CSS, cancer-specific survival; HR, hazard ratio; KSCC, keratinizing squamous cell carcinoma; M, distant metastasis; N, node; NKSCC, nonkeratinizing carcinoma; NPC, nasopharyngeal carcinoma; OS, overall survival; T, tumor. ^a^The 5-year OS and CSS for subjects of unknown race were 73.4% and 77.7%, respectively. ^b^T1, nasopharynx, oropharynx, or nasal cavity; T2, parapharyngeal extension; T3, bony structures and/or paranasal sinuses; T4, intracranial extension and/or cranial nerves, hypopharynx, orbit, or infratemporal fossa/masticatory space. ^c^N0, none; N1, unilateral cervical and/or unilateral or bilateral retropharyngeal node(s), ≤6 cm in greatest dimension, above supraclavicular fossa; N2, bilateral cervical node(s), ≤6 cm in greatest dimension, above supraclavicular fossa; N3, N3a: >6 cm in greatest dimension, above supraclavicular fossa; N3b: in supraclavicular fossa.

**Table 3:** Analysis of risk LN metastasis categories for distant metastasis using logistic regression in 1884 eligible NPC patients with LN metastasis.

LN metastasis category	Univariate analysis		Multivariate analysis	
	OR (95% CI)	*P*	OR (95% CI)	*P*
Level 2	Reference		Reference	
Levels 23	1.664 (0.926, 2.990)	0.089	1.714 (0.948, 3.100)	0.075
Level 1	2.065 (1.079, 3.950)	0.029	2.101 (1.090, 4.047)	0.027
Level 5	1.622 (0.802, 3.280)	0.178	1.506 (0.736, 3.084)	0.263
Levels 25	1.984 (1.019, 3.862)	0.044	1.949 (0.989, 3.843)	0.054
Levels 12	0.360 (0.085, 1.519)	0.164	0.356 (0.084, 1.513)	0.162
Levels 2345	4.295 (2.307, 7.999)	<0.001	4.064 (2.155, 7.666)	<0.001
Levels 235	0.994 (0.379, 2.608)	0.991	1.091 (0.413, 2.884)	0.861
Levels 2rp	1.458 (0.591, 3.596)	0.413	1.379 (0.552, 3.449)	0.492
Levels 234	1.215 (0.460, 3.209)	0.694	1.263 (0.476, 3.352)	0.639
Level rp	0.938 (0.278, 3.158)	0.917	0.960 (0.283, 3.254)	0.947
Level 3	1.382 (0.470, 4.065)	0.557	1.381 (0.464, 4.110)	0.562
Levels 23rp	1.930 (0.716, 5.206)	0.194	2.103 (0.772, 5.731)	0.146
Levels 24	2.853 (1.030, 7.906)	0.044	3.003 (1.074, 8.395)	0.036

CI, confidence interval; LN, lymph node; NPC, nasopharyngeal carcinoma; OR, odds ratio; rp, retropharynx. Level 2 means the patients with only level II LN metastasis. Levels 23 means the patients with level II and level III LN metastases, etc. Multivariate analysis adjusted for age, sex, and race.

**Table 4:** Cox regression analyses for evaluating the risk LN metastasis categories for CSS in 1884 eligible NPC patients with LN metastasis.

LN metastasis category	Univariate analysis		Multivariate analysis	
	HR (95% CI)	*P*	HR (95% CI)	*P*
Level 2	Reference		Reference	
Levels 23	1.106 (0.770, 1.587)	0.587	1.137 (0.786, 1.646)	0.495
Level 1	1.200 (0.811, 1.776)	0.361	1.175 (0.790, 1.749)	0.426
Level 5	1.073 (0.704, 1.634)	0.745	1.048 (0.682, 1.609)	0.832
Levels 25	1.397 (0.934, 2.090)	0.103	1.118 (0.736, 1.699)	0.600
Levels 12	1.361 (0.850, 2.180)	0.200	1.360 (0.841, 2.201)	0.210
Levels 2345	1.338 (0.844, 2.123)	0.216	0.928 (0.563, 1.528)	0.768
Levels 235	1.518 (0.989, 2.329)	0.056	1.708 (1.089, 2.678)	0.020
Levels 2rp	0.717 (0.365, 1.409)	0.334	0.588 (0.295, 1.173)	0.132
Levels 234	1.435 (0.876, 2.349)	0.151	1.299 (0.772, 2.188)	0.325
Level rp	1.106 (0.581, 2.105)	0.758	0.869 (0.452, 1.672)	0.674
Level 3	1.709 (1.017, 2.873)	0.043	1.602 (0.938, 2.737)	0.084
Levels 23rp	1.266 (0.665, 2.409)	0.472	1.248 (0.646, 2.408)	0.510
Levels 24	1.889 (1.045, 3.412)	0.035	1.587 (0.866, 2.908)	0.135

CI, confidence interval; CSS, cancer-specific survival; HR, hazard ratio; LN, lymph node; rp, retropharynx. Level 2 means the patients with only level II LN metastasis, Levels 23 means the patients with level II and III LN metastases, etc. Multivariate analysis adjusted for age, sex, race, histology, TNM stage, and treatment (radiotherapy, surgery, and chemotherapy).

## Data Availability

All data were acquired from the Surveillance, Epidemiology, and End Results (SEER) database. The data are available from the corresponding author upon request.
